# Clonal sector analysis and cell ablation confirm a function for *DORNROESCHEN-LIKE* in founder cells and the vasculature in *Arabidopsis*

**DOI:** 10.1007/s00425-020-03545-5

**Published:** 2021-01-08

**Authors:** Dorothea Glowa, Petra Comelli, John W. Chandler, Wolfgang Werr

**Affiliations:** Developmental Biology, Institute of Zoology, Cologne Biocenter, Cologne University, Zülpicher Straße 47b, 50674 Cologne, Germany

**Keywords:** Cell ablation, Diphtheria toxin A, DORNRÖSCHEN-LIKE, Founder cells, Sector analysis, Transcription factor

## Abstract

*****Main conclusion***:**

**Inducible lineage analysis and cell ablation via conditional toxin expression in cells expressing the DORNRÖSCHEN-LIKE transcription factor represent an effective and complementary adjunct to conventional methods of functional gene analysis.**

**Abstract:**

Classical methods of functional gene analysis via mutational and expression studies possess inherent limitations, and therefore, the function of a large proportion of transcription factors remains unknown. We have employed two complementary, indirect methods to obtain functional information for the AP2/ERF transcription factor DORNRÖSCHEN-LIKE (DRNL), which is dynamically expressed in flowers and marks lateral organ founder cells. An inducible, two-component Cre–Lox system was used to express beta-glucuronidase GUS in cells expressing *DRNL*, to perform a sector analysis that reveals lineages of cells that transiently expressed *DRNL* throughout plant development. In a complementary approach, an inducible system was used to ablate cells expressing *DRNL* using diphtheria toxin A chain, to visualise the phenotypic consequences. These complementary analyses demonstrate that *DRNL* functionally marks founder cells of leaves and floral organs. Clonal sectors also included the vasculature of the leaves and petals, implicating a previously unidentified role for DRNL in provasculature development, which was confirmed in cotyledons by closer analysis of *drnl* mutants. Our findings demonstrate that inducible gene-specific lineage analysis and cell ablation via conditional toxin expression represent an effective and informative adjunct to conventional methods of functional gene analysis.

## Introduction

Conventional genomic approaches to identify gene functions include assessing the genetic and phenotypic consequences of gene loss-of-function or overexpression, coupled with detailed tissue-specific expression analysis. However, these methodologies are often limited by genetic redundancy and subtle or transient phenotypes. Furthermore, dynamic spatio-temporal expression patterns are difficult to capture by in situ hybridisation, and reporter gene analysis and live imaging depend on using complete regulatory sequences to recapitulate native expression. This means that a function can still not be ascribed to a large proportion of *Arabidopsis* genes.

To overcome these limitations, sector analysis in parallel with the genetic ablation of cells within a specific gene expression domain allows domains of gene expression to be visualised indirectly in individual cells or tissue by analysing the derived cell lineages or the developmental consequence of loss of the expression domain at a specific time point. The power of these approaches resides in the ability to reveal spatiotemporally transient or subtle expression domains and developmental consequences that might not be evident by the conventional methods.

Clonal sector analysis has a long tradition in plant research and has enabled many outstanding questions in developmental biology to be elucidated. For example, establishing the number of organ founder cells (Poethig and Sussex 1985; Bossinger and Smyth 1996), creating tissue fate maps (Irish and Sussex 1992; Woodrick et al. 2000), or analysing stomatal cell lineages (Serna et al. 2002). In other contexts, sectors have been created conditionally or induced tissue-specifically (Sieburth et al. 1998; Saulsberry et al. 2002; Wachsman et al. 2011).

Cell or tissue ablation has also been applied within many contexts of plant developmental biology. Physical methods such as micro-dissection or laser ablation have revealed the embryogenic potential of the suspensor (Liu et al. 2015) and informed models of phyllotaxy (Reinhardt et al. 2005). Tissue- or cell-type-specific cell ablation has been targeted by conditionally expressing the diphtheria toxin A chain (DT-A)-coding sequence via promoters such as *APETALA3* (Day et al. 1995), the *AGAMOUS* intron (Liu and Liu 2008), *LEAFY* (Nilsson et al. 1998), the *S Locus Glycoprotein* gene (Kandasamy et al. 1993; Thorsness et al. 1993), or embryo-specific promoters (Weijers et al. 2003). Genetic ablation of the root cap via DT-A has also elucidated its role in root meristematic activity and lateral root initiation (Tsugeki and Federoff 1999).

The APETALA2/ETHYLENE RESPONSE FACTOR transcription factor DORNRÖSCHEN-LIKE (DRNL) has been well characterised functionally in *Arabidopsis* in different developmental contexts, including embryonic patterning, floral meristem identity, and floral organ initiation (Chandler et al. 2007; Chandler and Werr 2017), where it functions redundantly with its paralogue DORNRÖSCHEN (DRN) or related protein PUCHI. The dynamic expression pattern of *DRNL* in prepatterning outer floral organ whorls and subsequently in marking groups of cells that correspond spatiotemporally and numerically with the earliest stages of floral organ initiation (Chandler et al. 2011b) has implicated it as a founder-cell marker. However, partly due to redundancy, loss of *DRNL* function leads to relatively subtle phenotypes. A weak allele shows stamen fusion at low penetrance (Chandler et al. 2011b), and in a strong allele, stamen outgrowth is inhibited and first-whorl floral organs adopt a sepal–petal mosaic identity (Nag et al. 2007).

Although the expression domains of the *DRNL* gene and protein are coincident (Chandler et al. 2011a), discrepancies exist between these domains and loss-of-function phenotypes. For example, *DRNL* contributes to cell-division planes in the basal embryo domain, although it is only expressed in the apical embryo domain from late globular stages onwards (Chandler et al. 2007) and it contributes to the acquisition of floral meristem identity, despite not being expressed within the central zone of the floral meristem (Chandler and Werr 2017). These potentially cell non-autonomous functions of *DRNL*, together with potential limitations in the resolution of the *DRNL* expression domains provided by current reporter systems and the presence of functions masked by genetic redundancy prompted us to investigate the fate of clonally derived cells that express *DRNL*, both visually via sector analysis, and functionally by their genetic ablation. We have generated β-glucuronidase (GUS) sectors by producing CRE recombinase in *DRNL*-derived lineages using a dexamethasone-inducible system (Metzger and Chambon 2001; Samalova et al. 2019), or we have ablated cells that express *DRNL* with diphtheria toxin A (DT-A) (Breitmann et al. 1987; Weijers et al. 2003). In addition to confirming the efficacy of sector and ablation analysis for functional gene analysis, the specific aims of this study were to functionally address the hypothesis that *DRNL* marks floral organ founder cells, to potentially reveal novel expression domains not hitherto identified, and to focus on *DRNL* function in leaves, which show no mutant phenotype, despite expression of *DRNL* in their primordia.

The pattern of GUS sectors was largely corroborated by ablation phenotypes and the absence of floral organs and leaf primordia functionally confirmed that *DRNL* marks founder cells. In addition, GUS expression and ablation phenotypes identified *DRNL* expression in the leaf vasculature that has previously not been described, and cotyledon vascular defects were observed in two *drnl* mutant alleles. The data emphasise the utility of these methodologies to extend knowledge of specific plant gene functions.

## Materials and methods

### Clonal analysis and ablation constructs

**Fig. 1:**
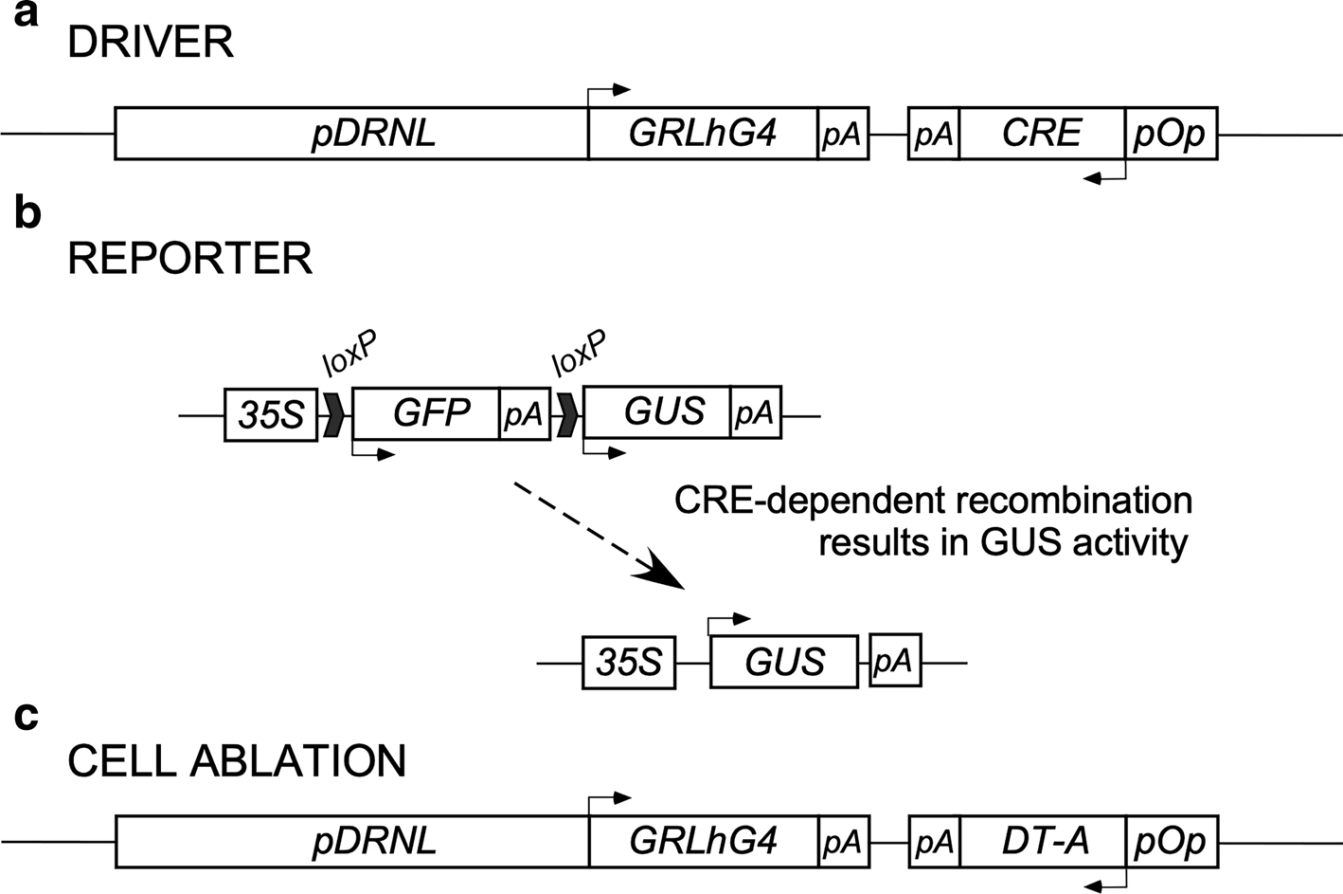
Schematic presentation of driver, reporter, and cell-ablation constructs. **a** The *DRNL* driver cassette consists of 5644 bp of upstream promoter sequence, the *DRNL* transcription start, and 5**′** untranslated region (UTR) in front of the GRLhG4-encoding region (Craft et al. 2005). The chimeric GRLhG4 transcriptional activator accumulates in the cytoplasm until dexamethasone (DEX) enables nuclear import. In the nucleus, GRLhG4 binds to the *pOp* promoter and induces transcription of the *CRE*-recombinase gene. **b** After translation, the CRE-recombinase recognises *loxP* sites (indicated by bold arrows) that flank the *GFP* transcription unit as direct repeats. The CRE-dependent excision of the GFP cassette results in the expression of the GUS marker behind the constitutive *CaMV* 35S promoter. **c** For cell ablation, the CRE-recombinase-encoding region in the driver construct (**a**) was replaced by the diphtheria-A toxin open-reading frame, which is expressed in cells expressing *DRNL* following DEX application

To visualise the fate of cells that transiently express the *DRNL* founder-cell marker in the SAM peripheral zone, we combined the *DRNL* promoter with a dexamethasone (DEX)-inducible chimeric GRLhG4 transcriptional activator (Craft et al. 2005). The GRLhG4 transcription activator is a fusion between the hormone-binding domain of the rat glucocorticoid repressor and the previously described high‐affinity DNA‐binding domain mutant of the LAC repressor (Lh), and the transcription‐activation‐domain‐II of GAL4 (G4) from *Saccharomyces cerevisiae* (Moore et al. 1998). When cytoplasmic retention of GRLhG4 is released by DEX application, the transcriptional activator binds to the chimeric promoter *pOp* that consists of ideal lac operators in front of the minimal CaMV 35S promoter (Moore et al. 1998; Craft et al. 2005). As a driver (Fig. 1a), we combined the *DRNL::GRLhG4* transgene with a *pOp::CRE-*recombinase target gene within the same T-DNA construct. To monitor *DRNL* promoter-dependent CRE-recombinase activity, we used activation of the β-glucuronidase (GUS) marker gene behind the constitutive *CaMV* 35S promoter following *in planta* excision of a GFP cassette in front of the GUS coding region (Fig. 1b). To compare GUS-stained clonal sectors with the effects of developmentally induced cell ablation, we also used the *DRNL::GRLhG4* transgene to conditionally express the diphtheria-A-toxin following DEX treatment in a cell-type-specific manner (Fig. 1c).

*GRLhG4* was transcribed in lateral organ founder cells using 5644 bp upstream *DRNL* sequence extending from the upstream *At1g24610* stop codon to the *DRNL* (*At1g24590*) translation start codon and replacing the *GFP* coding region of the *DRNL::GFP* marker (Chandler et al. 2011b) by the *GRLhG4* (Craft et al. 2005) open-reading frame, which required conversion of a unique *Xma*I site into a *StuI* site. The *CRE*-recombinase coding region was isolated from the *pCRE-GR* plasmid (Metzger and Chambon 2001) and combined with the *pOp* target promoter (Craft et al. 2005). Cloning was performed following standard procedures (Green and Sambrook 2012) and involved intermediate subcloning steps in different pRT expression cassettes (Töpfer et al. 1987; Überlacker and Werr 1996), which either directly provided the 3′-terminal polyadenylation signal (polyA) or unique restrictions sites for insertion of PCR fragments. The PCR primers flanking the *GRLhG4* and *CRE* genes are listed in Table 1, and in the final driver construct, the *CaMV* 35S polyA signal was inserted behind the *GRLhG4* open-reading frame (ORF) or the *Agrobacterium tumefaciens* octopine synthase (*ocs*) polyA signal behind the *CRE* coding region. The bacteriophage P1 Cre–Lox recombination system acts efficiently in plants (Gilbertson 2003) and the *DRNL::GRLhG4*-*pOp::CRE* driver allows spatial and temporal control: first, the *DRNL* promoter spatially specifies *GRLhG4* transcription, and second, the expression and activity of CRE recombinase depends on the glucocorticoid dexamethasone (DEX), which allows GRLhG4 to enter the plant cell nucleus and to activate *CRE* transcription. For cell ablation, the *CRE* coding region was replaced by the diphtheria toxin A chain ORF (Breitmann et al. 1987) in the *DRNL::GRLhG4*-*pOp::DT-A* ablation construct, by blunt-ending the unique *Nde*I in front of the *CRE* ORF and using an *Xba*I site downstream and preceding the *ocs* polyA signal.

**Table 1:** Primers used to amplify the *GRLhG4*, *CRE,* and *DT-A* open-reading frames

Construct	Plasmid	Sequence (5′–3′)
*DRNL::GRLhG4*	StuI-GRLhG4_F	AGGCCTATGGCTAGTGAAGCTCGAAAAAC
	StuI-GRLhG4_R	AGGCCTCATTTTACTCTTTTTTTGGGT
*pOp::CRE*	NdeI_Cre_F	CATATGTCCAATTTACTGACC
	Cre_TAG_R	CTAATCGCCATCTTCCAGCAGG
*pOp::DT-A*	DTA_ATG_F	ATGTTGTTGATTCTTCTAAATC
	XbaI_DTA_R	TCTAGATCGCCTGACACGATTTCCTG

The basis for the GUS reporter construct (Fig. 1b) was an existing cassette of the erGFP marker between the *CaMV* 35S promoter and the 35S polyA signal in the pRTΩ/*Not*I *Asc*I vector (Überlacker and Werr 1996). A unique *Xba*I site between *GFP* ORF and its 35S polyA terminus was used to insert the nopaline synthase polyA signal followed by a *loxP* site in front of the complete *uidA* (GUS)-coding region. The second *loxP* site was inserted at the 5′ terminus of the *GFP* ORF by directional cloning into unique *Not*I and *Nco*I sites; two *Asc*I sites at the outer flanks of the excision cassette were subsequently used to transfer the reporter construct into the binary *pGPTV-AscI-KAN* vector (Überlacker and Werr 1996). The *pGPTV-AscI-BAR* vector was used as a recipient for the *DRNL::GRLhG4*-*pOp::CRE* driver and the *DRNL::GRLhG4*-*pOp::DT-A* cell-ablation constructs. The *DRNL::GUS* transgene was constructed by replacing the *DRNL*-encoding sequences by the *GUS* open-reading frame in the context of flanking *DRNL* genomic sequences extending to the upstream and downstream genes, analogous to the *DRNL::GFP* reporter (Chandler et al. 2011b).

All constructs were introduced into *Agrobacterium tumefaciens* GV3101, which was used for the transformation of *Arabidopsis thaliana* Col by the floral dip method (Bechthold and Pelletier 1998).

### Plant material and growth conditions

Transgenic progeny were selected via resistance to Kanamycin (Thermo Fisher Scientific, Dreieisch, Germany) or the herbicide BASTA (glufosinate ammonium, Thermo Fisher Scientific) and lines carrying single transgene copies were identified based on a 3:1 segregation of resistance markers. The different resistance markers of driver and reporter lines were used to select double-transgenic lines after crosses; however, results for these lines did not differ substantially from those of lines generated by cotransformation with the driver/reporter constructs followed by direct double selection for Kanamycin^R^ and BASTA^R^. At least six independent driver/reporter combinations that originated from single transformations and crosses, or double transformations/selections were analysed, but because all lines behaved similary, we focussed on single stable reporter/driver combinations or *DT-A* lines to characterise either the sector patterns or ablation phenotypes, respectively. To monitor early events, seeds were fume-sterilised in a sealed container with 3% hydrochloric acid for 2 h and germinated on Murashige and Skoog (1962) medium on plates supplemented with 5 µg mL^−1^ DEX. Alternatively, lines were grown on soil and sprayed with DEX at the fully open cotyledon stage or prior to bolting at the 6–7 leaf stages (Boyes et al. 2001). To assess embrogenic phenotypes with the ablation construct, primary inflorescences were sprayed with DEX at the time of first flower opening, and seeds were harvested from the first 5–6 siliques for the analysis of growth defects. All seeds were stratified for 2 days in a 4 °C dark room and grown in long-day conditions (16 h light/8 h dark). The *drnl-1* and *drn-2* mutants have been described previously (Chandler et al. 2007) as has *pid-2* (Chandler et al. 2011a).

### GUS staining and imaging

Histological analysis and GUS staining essentially followed the protocol of Blazquez et al. (1997), and seedlings, whole plants, or primary inflorescences were incubated in 50 mM X-gluc staining solution, 50 mM sodium phosphate buffer pH 7, 0.2% Triton X-100, 3 mM potassium ferricyanide, 3 mM potassium ferrocyanide, 20% methanol, first on ice under vacuum for 15 min, then at 37 °C until an optimal signal-to-noise ratio was observed under the dissecting microscope (8–16 h). For whole-mount images, tissue was destained and dehydrated in a series of 50, 70, and 100% ethanol, and rehydrated in a reverse series before microscopical analysis. Images were captured on a Leica MZ6 or MZ16FA dissecting microscope and processed or assembled using the Adobe Photoshop (version 21) software.

## Results

### Clonal sectors during seedling and early vegetative development

**Fig. 2:**
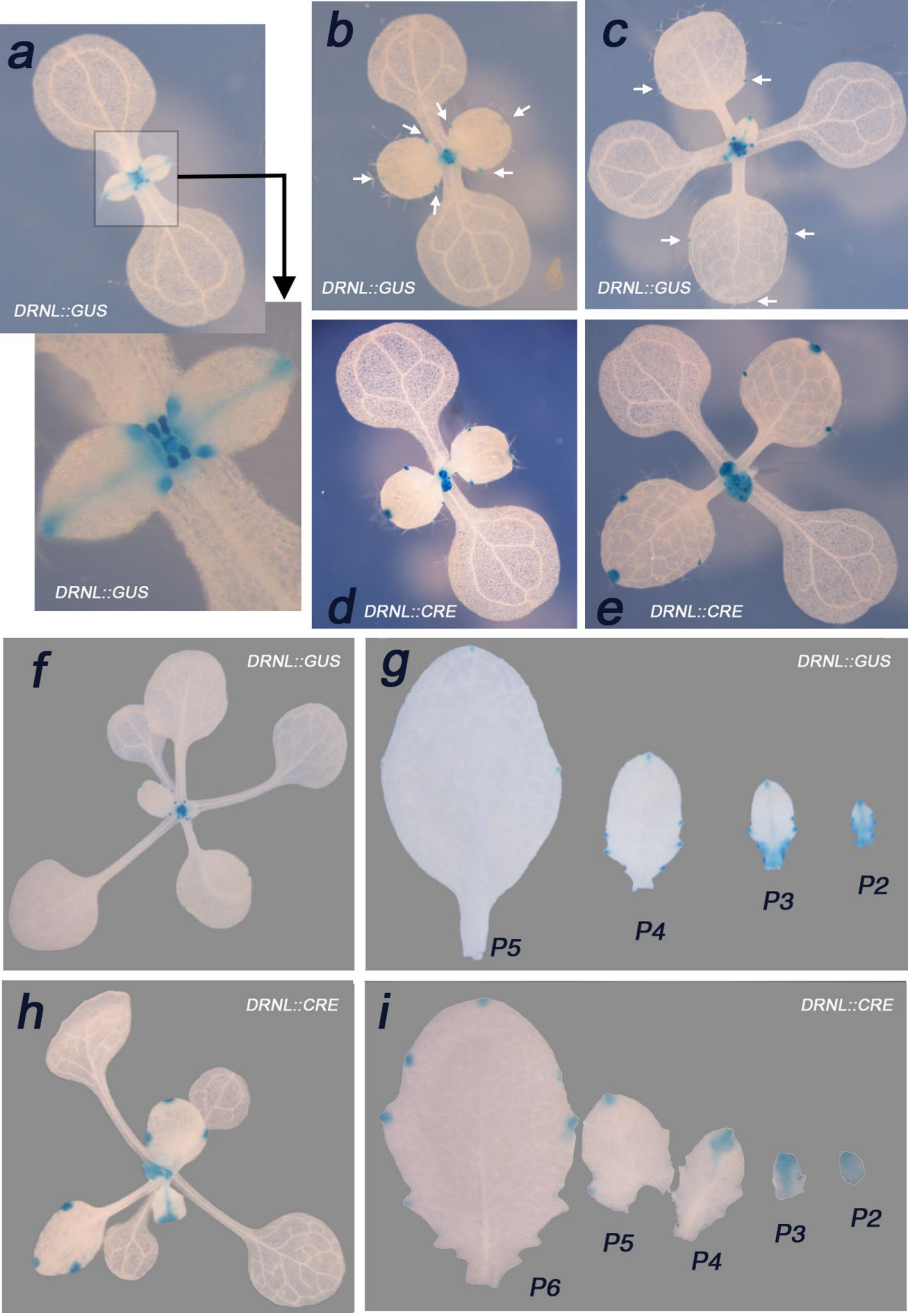
Differences between *DRNL::GUS* promoter activity and clonal GUS sectors due to DEX-dependent *DRNL::CRE* activity in driver/reporter combinations. The relevant transgene *DRNL::GUS* or *DRNL::CRE* is indicated on the individual pictures. **a** Young *DRNL::GUS* seedling stained for GUS activity at the two-leaf stage. Note the absence of detectable GUS activity in the cotyledons but staining in the developing leaf primordia, which are depicted in a close-up below. The two initial leaves are at a similar developmental stage and show *DRNL::GUS* expression at the apical tip of the leaf lamina that extends through the mid-rib towards the lamina base, where it is most prominent in two lateral foci, that probably represent developing serrations or hydathodes. At the base of each petiole, which is still not elongated, GUS activity marks pairs of stipules. **b**, **c** *DRNL::GUS* expression in slightly older seedlings: GUS activity at the margins is marked by arrows in leaves 1 and 2 decreases at the apical lamina tip, but is still visible in two lateral foci, whereas GUS expression is strong in the young leaf primorida 3 and 4 (**b**). In an older seedling (**c**)**,** the GUS expression pattern in leaves 3 and 4 is the same as that in younger leaves 1 and 2 (close-up in **a**). **d**, **e** The clonal GUS expression pattern in driver/reporter combination lines when seeds are germinated in the presence of DEX. The developmental stages are comparable to those depicted in **b** or **c**. Note the prominent apical sector and the basal hydathode sectors in leaves 1 and 2 and the very strong staining of leaves 3 and 4 in **d**, a pattern that remains unaltered in the slightly older seedling (**e**). Compare the completely stained leaves 3 and 4 in **e** to the local *DRNL::GUS* pattern in **c**. The differences between the GUS-staining pattern in *DRNL::GUS* marker lines and DEX-dependent clonal sectors in driver/reporter combinations are apparent in whole-seedling top views (**f**, **h**) and detached leaves (**g**, **i**). **f** Top view of a *DRNL::GUS* seedling with GUS expression in the youngest leaf primordia close to the SAM. **g** Dissected leaves of a *DRNL::GUS* plantlet showing small-expression domains associated with the hydathodes in maturing leaves (P4, P5), accompanied by some basal staining in leaf P3 and the mid-rib in P2. **h**, **i** Clonal sectors in driver/reporter combinations following spraying of seedlings with DEX at the fully opened cotyledon stage. **h** Whole seedling with sectors in leaves 3–6 and possibly 7; note the small sectors at the apical tip and at two lateral positions in leaves 3 and 4 relative to the larger wedge-shaped GUS domains in leaf 5 or the entire leaflet 6. **i** Dissected leaves 3**–**7 of a seedling similar to that in **h**; note the size of lateral hydathode sectors in leaf 5 (P6) relative to the small signals in leaf 4 (P5) of a *DRNL::GUS* plantlet in **g** and the increasing size and wedge shape of the apical sectors in younger leaflets relative to the dominance of lateral sectors in older leaves. The background in images **f**–**i** has been adjusted using Adobe Photoshop

To compare cellular *DRNL* promoter activity with clonal sectors that reflected cell divisions after DEX induction and CRE-dependent activation of the *35S::GUS* reporter, we analysed GUS expression in plants containing the *DRNL::GUS* transgene or DEX-treated driver/reporter combination lines at similar developmental stages (Fig. 2). CRE-dependent recombination was confirmed by PCR (data not shown) in genomic DNA from blue-sectored organs, whereas DNA from white explant control tissue only contained the non-recombined reporter constructs.

The *DRNL* promoter was active at the leaf tip (Fig. 2a) and throughout the mid-rib to the lamina base, where expression focussed on two lateral marginal foci and was strong in stipules at the base of the petiole. This expression pattern was recapitulated in subsequently formed young leaves (e.g., leaves three and four), whereas *DRNL::GUS* expression in older leaves became restricted to weak foci at the apical and lateral margins (see arrows in Fig. 2b, c).

By contrast, DEX-induced clonal GUS sectors in leaves three and four were broader than domains of *DRNL::GUS* expression and encompassed the entire leaf lamina (Fig. 2d, e), and were larger in older leaves one and two when *DRNL::GUS* activity was already very weak (compare Fig. 2c, e). Differences in *DRNL::GUS* and DEX-induced *35S-GUS* expression were also observed in older seedlings, which expressed *DRNL::GUS* in the newest initiated leaves (Fig. 2f), whereas DEX-induced GUS sectors (Fig. 2h) were present in the earliest leaf primordia and extended into successive maturing leaves. Marginal GUS sectors in leaves three and four relate to *DRNL::GUS* activity in the hydathodes, and staining in leaves five and six correspond to *DRNL* expression in early leaf development. GUS sectors became visible after a time-delay following DEX induction, but extrapolation to native *DRNL* expression domains supports the hypothesis that inducible *DRNL::CRE*-recombinase activity activates the *35S::GUS* reporter, which is transmitted cell-autonomously by cell divisions and describes clonal cellular developmental trajectories.

To address the delay between *DRNL* promoter activity and the generation of clonal sectors, we compared *DRNL::GUS*-stained leaves at different developmental stages, except for P0 and P1, due to their inaccessibility at the SAM. *DRNL::GUS* activity was detected in P2–P5 leaves at the apical tip, the mid-rib and at the base of P2 leaflets (Fig. 2g). In P3 leaves, *DRNL::GUS* expression was present at the apical tip, the mid-rib, the base of the lamina in the petiole, and leaf serrations and/or hydathodes. In P4 leaves, *DRNL* promoter activity was restricted to the tip of the mid-rib and hydathodes—local domains that were further reduced in P5 leaves. By contrast, clonal GUS sectors (Fig. 2i) preferentially marked the apical domain of P2 leaves, with a wedge-shaped GUS-staining pattern that was more pronounced in P3 leaves. An apical sector that tapered into the mid-rib was detected in P4 leaves and small domains of GUS expression remained in the apical part of P5/P6 leaves and at the tip of serrations, or were associated with hydathodes and were larger than the extremely small *DRNL::GUS* expression foci observed at this developmental stage.

### Clonal sector variability in vegetative rosette leaves

**Fig. 3:**
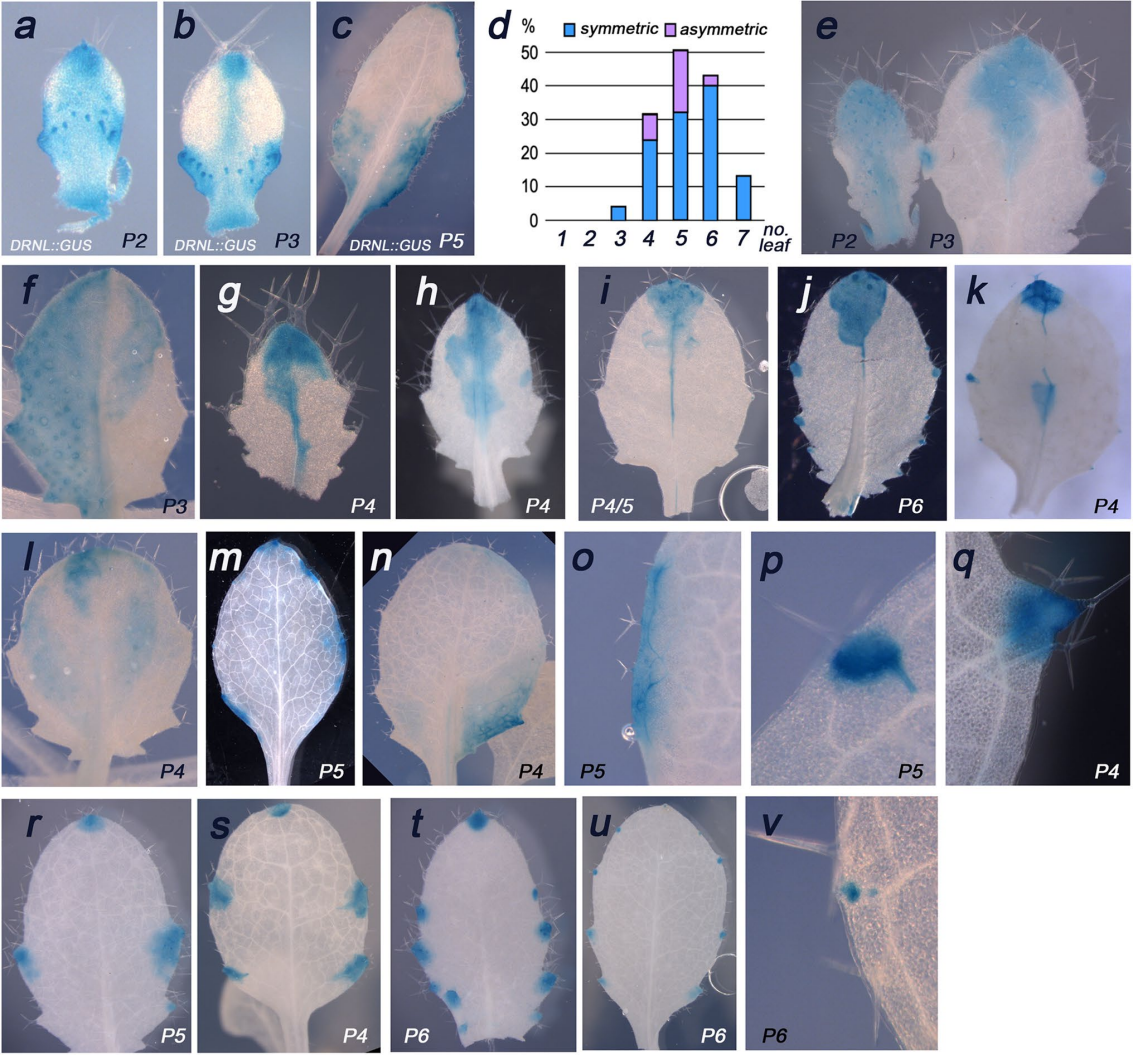
Differences between promoter activity and clonal sectors in leaves. **a**–**c** Dynamic expression pattern of the *DRNL::GUS* transgene as indicated on each picture for young leaf stages. **d** Sector distribution in seedlings expressing driver/reporter combinations sprayed with DEX when the cotyledons were fully opened. GUS sectors were absent from the two first leaves initiated in the embryo, and according to the biological half-life and threshold levels of DEX, DEX-dependent sectors were restricted to the next five successive leaves. Numerous sectors were observed in leaves 4–6, of which a substantial fraction (in purple) exhibited asymmetric apical domain sectors. All subsequent panels depict DEX-dependent clonal sectors due to *DRNL::CRE-GR* activity in driver/reporter combinations. **e** Two successive young leaves from the same seedling with representative clonal sectors in young leaf primordia. Note that in contrast to the *DRNL::GUS* pattern in **a** or **b,** the lateral lamina domains are free of GUS staining, whereas staining that includes the mid-rib in the younger or the apical sector and hydathodes in the older leaflet coincide with *DRNL* promoter activity. **f**–**k** A selection of asymmetric sectors and independent cellular trajectories in lamina halves. **f** Half-lamina early sector along the mid-rib (left) and an independent apical sector (right); **g** mid-rib sector with differential expansion in the apical domain; **h** differently shaped sectors on either side of the mid-rib; note the absence of expression in a small central region to the left of the mid-rib and the sector at the leaf margin to the right. **i** Split mid-rib sector and discrete GUS-stained cellular trajectories to either side of the mid-rib below a symmetrical wedge-shaped apical sector. **j** An asymmetric apical sector that in contrast to sectors in **i** or **k** includes the epidermal layer and hydathode sectors at either margin. **k** A split mid-rib sector that centrally extends into the left lamina half; note the GUS staining in secondary veins in the apical domain. **l**–**o** Less-frequent sectors observed in basal lamina domains, which potentially relate to transient *DRNL* promoter activity during early leaf development (compare with *DRNL::GUS* patterns in **a**–**c**. **p** Close-up of a hydathode sector that includes the terminal part of the connected vein. **q** Sector that includes a leaf serration. **r**–**u** Hydathode-associated sectors with increasing leaf age from **r** to **u** at the time of DEX application; note the absence of an apical sector and small sector size (**u**). **v** Two distinct clonal sectors associated with a single hydathode suggest independent cellular events

The size and shape of apical *DRNL::GUS* sectors in leaves varied during early leaf development (Fig. 3a–c). When clonal GUS sectors in driver/reporter lines were analysed 7 days following DEX application, a single DEX pulse typically resulted in up to five sectored leaves (Fig. 3d) and allowed the earliest recombination events in newly initiated P0 or P1 primordia and in more developed leaflets to be visualised.

The largest GUS sectors relative to leaf size were reproducibly observed in the youngest two successive leaves (Fig. 3e). Although the apical wedge-shaped sectors often present in P2 leaves covered most of the leaf, those in P3 leaves were smaller. However, sector size was highly variable among individual plantlets and relates to *DRNL* promoter activity relative to leaf developmental stage, to the number of cells that underwent CRE-recombination, or to temporal differences in the course of the DEX pulse. Sector shape is more relevant than sector size, because GUS staining reflects the position of cells that underwent recombination and reveals the fate of these cells during subsequent leaf development. Shape asymmetries are particularly informative, because these depict cellular trajectories in the same leaf developmental window and under similar conditions of CRE-recombinase activity. Asymmetric sectors between left and right lamina halves accounted for up to 20% of apical sectors (Fig. 3d), but were also observed at the lamina margins. Sectors were rarely split by the mid-rib (Fig. 3f), and these reflect independent cellular trajectories within each lamina half. The blue-stained entire left leaf half probably represents a recombination event early during leaf initiation, potentially in few P0 cells, whereas the isolated apical sector in the right lamina half depicts activation of the GUS reporter that occurred substantially later. Other staining asymmetries (Fig. 3g–k) varied with respect to the extension of the sector towards the leaf base, broadness, and gaps. The sharpness of some clonal boundaries (Fig. 3j) reflects the inclusion of the epidermal layer.

Fewer sectors were observed in basal or lateral leaf domains than in the apical domain. Some medial lamina domain sectors originated simultaneously with apical sectors (Fig. 3l), whereas others were confined to the margins (Fig. 3m) or to the base (Fig. 3n). Staining of the vasculature was associated with sectors that included the hydathodes (Fig. 3p, q), mainly in older leaves. Clonal sectors that included the hydathodes were observed at various developmental stages and in young leaflets were often combined with a large apical GUS sector (Fig. 3j, k), whereas only a small GUS-positive sector was detected at the tip of the mid-rib in older leaf primordia. The hydathode-associated sectors were larger in younger leaves than in older leaves (Fig. 3r–u), where additional sectors were present at the lamina base (Fig. 3s, t). Furthermore, hydathode-associated sectors in mature leaves were larger at the lamina base than those more apical (Fig. 3u). Noticeably, however, such GUS-stained sectors might not derive from CRE-dependent activation in single cells, but depict concerted recombination events in multiple cells (Fig. 3v).

### Floral organ clonal sectors

**Fig. 4:**
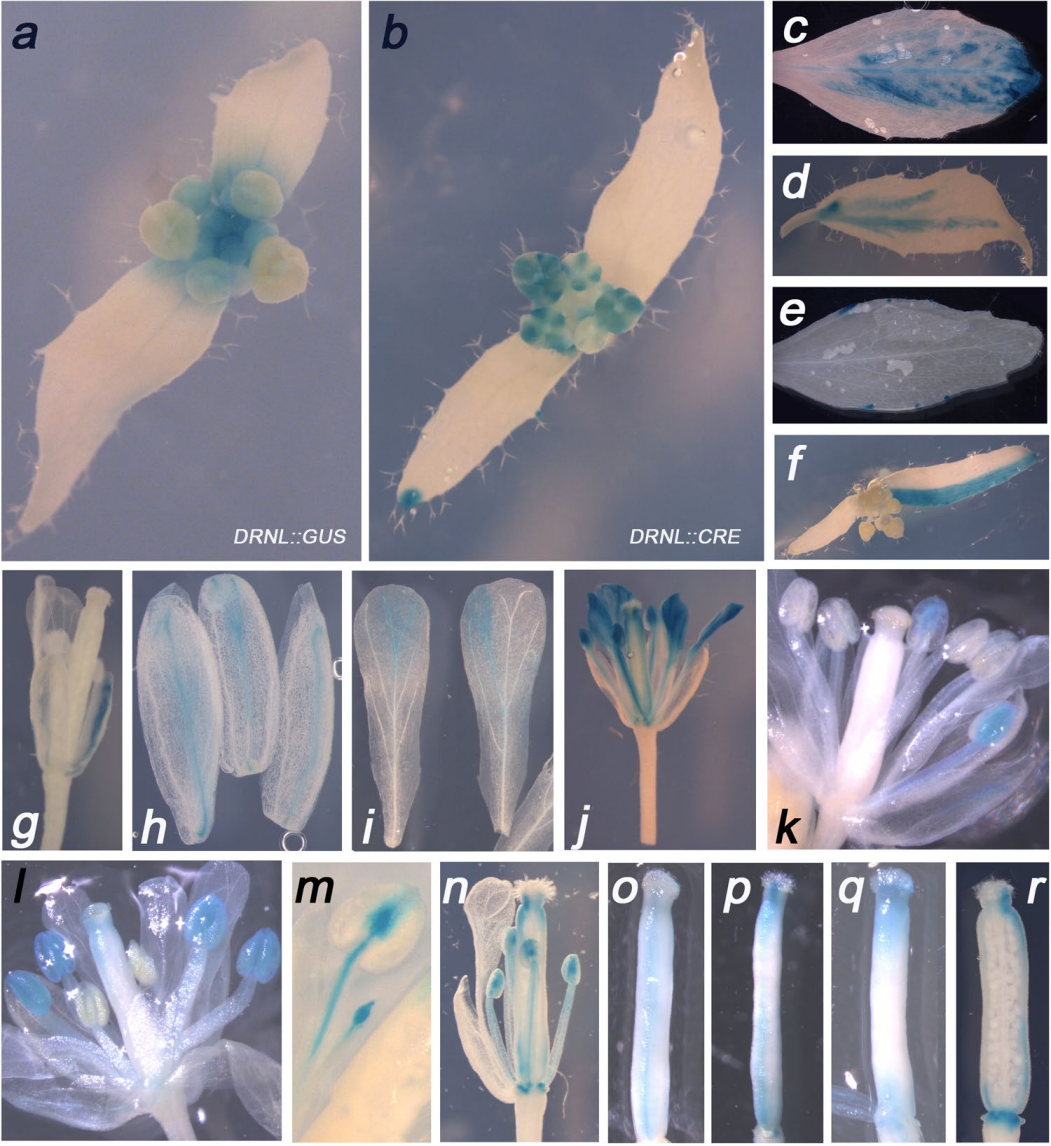
Clonal sector analyses in the inflorescence and flowers. **a**, **b** Top-view comparison between a *DRNL::GUS* inflorescence depicting transcriptional activity of the *DRNL* promoter (**a**) and *DRNL::CRE*-induced clonal sectors in an inflorescence of a driver/reporter combination line after DEX treatment (**b**). Note the local GUS staining of sepals and the apical GUS sector in the lower cauline leaf relative to the diffusely GUS-staining *DRNL::GUS* inflorescence and the non-stained floral buds in **a**. All subsequent panels depict DEX-dependent *DRNL::CRE-GR* activity in driver/reporter combinations, i.e., clonal sectors. **c**–**f** A selection of representative clonal sectors in cauline leaves that show a wedge-shaped apical preference (**c**), basal trajectories (**d**), specificity for the hydathodes (**e**), or a large stripe along one lamina of a single cauline leaf (**f**). **g**–**s** Sectors in floral organs: **g**, **h** typical sepal sectors along the mid-rib that can affect single or multiple sepals; **i**, **j** petal sectors are characteristically wedge-shaped, apically localised, and frequently coincide with stamen sectors. **k**, **l** *DRNL* promoter activity temporally differs among lateral and medial stamens, and sectors can be selectively present within either both lateral (**k**) or the four medial stamens (**l**). Clonal sectors can be restricted apically (**m**) or extend to the base of the filament (**n**) and medial sepal sectors often are accompanied by sectors in the gynoecia (**o**–**r**). Sectors along the replum are frequent, where both carpels are fused (**o**), and in the apical domain (**p**, **q**). Staining at the carpel base (**r**) potentially relates to late *DRNL* promoter activity in stamen-associated nectaries

Differences were also observed between *DRNL::GUS* expression and DEX-dependent clonal sectors during the reproductive stage (compare Fig. 4a, b). In young inflorescences, *DRNL:GUS* staining was strong in young buds and the centre of the inflorescence (Fig. 4a). By contrast, clonal sectors were observed at the tips of cauline leaves and in older flowers, but were hardly detectable in the inflorescence centre (Fig. 4b). The wedge-shaped, disparate cauline-leaf-staining-pattern in Fig. 4c represents multiple independent clonal trajectories and involved the vascular network, as does the basal sector in Fig. 4d. Sectors were associated with hydathodes at the cauline leaf margins (Fig. 4e) or were bisected by the mid-rib (Fig. 4f). Cauline leaves and vegetative leaves thus shared similar clonal patterns, except that the lanceolate shape of cauline leaves resulted in longer proximo-distal sectors.

A single DEX pulse typically generated GUS-stained sectors in 4–6 flowers. In individual inflorescences, the organ specificity of sectors inversely followed the sequence of organ initiation during floral development in successive flowers. Flowers that were most advanced at the start of the DEX pulse were located at the most basal stem positions and preferentially contained sectors in carpels and stamens. By contrast, sectors in petals or sepals originated later, but remained detectable in flowers that were youngest at DEX treatment. Because the softness of the tissue after GUS staining hampered the separation of organs from individual flowers to document the histology of sectors, and due to the dynamic *DRNL* expression pattern within the early window of lateral organ founder-cell specification, we restricted comparisons to elaborated floral organs.

Floral GUS sectors were often exclusive to individual sepals (Fig. 4g) and sectors generally involved the mid-rib and mostly extended from the apex to the base (Fig. 4h). Mature sepals and petals showed weak GUS staining, potentially reflecting weak *CaMV* 35S promoter activity. The flower in Fig. 4i shows the absence of GUS signal in sepals, but strong expression in the three inner floral organ whorls. DEX-dependent clonal sectors in petals were usually wedge-shaped and apical (Fig. 4j), whereas those in stamens were often specific to the anthers of both lateral stamens (Fig. 4k) or marked the four medial stamens and the style (Fig. 4l). In stamens, GUS sectors often included the connective tissue of the anthers and the vasculature of the filament, which was either completely or only apically stained, similar to patterns in the leaf or sepal mid-rib (Fig. 4m, n).

In carpels, GUS-stained clonal sectors were either narrow and extended along the entire gynoecium in a stripe (Fig. 4o), or marked domains at the base and the tip (Fig. 4p, q), occasionally connected by a stripe (Fig. 4p). The most frequent staining pattern was GUS sectors at the apical tip of the style and basal gynoecium sectors, with the absence of staining in internal gynoecium tissue and the developing ovules (Fig. 4r).

### Diphtheria toxin A ablation phenotypes are consistent with *DRNL* promoter activity and clonal GUS sectors

**Fig. 5:**
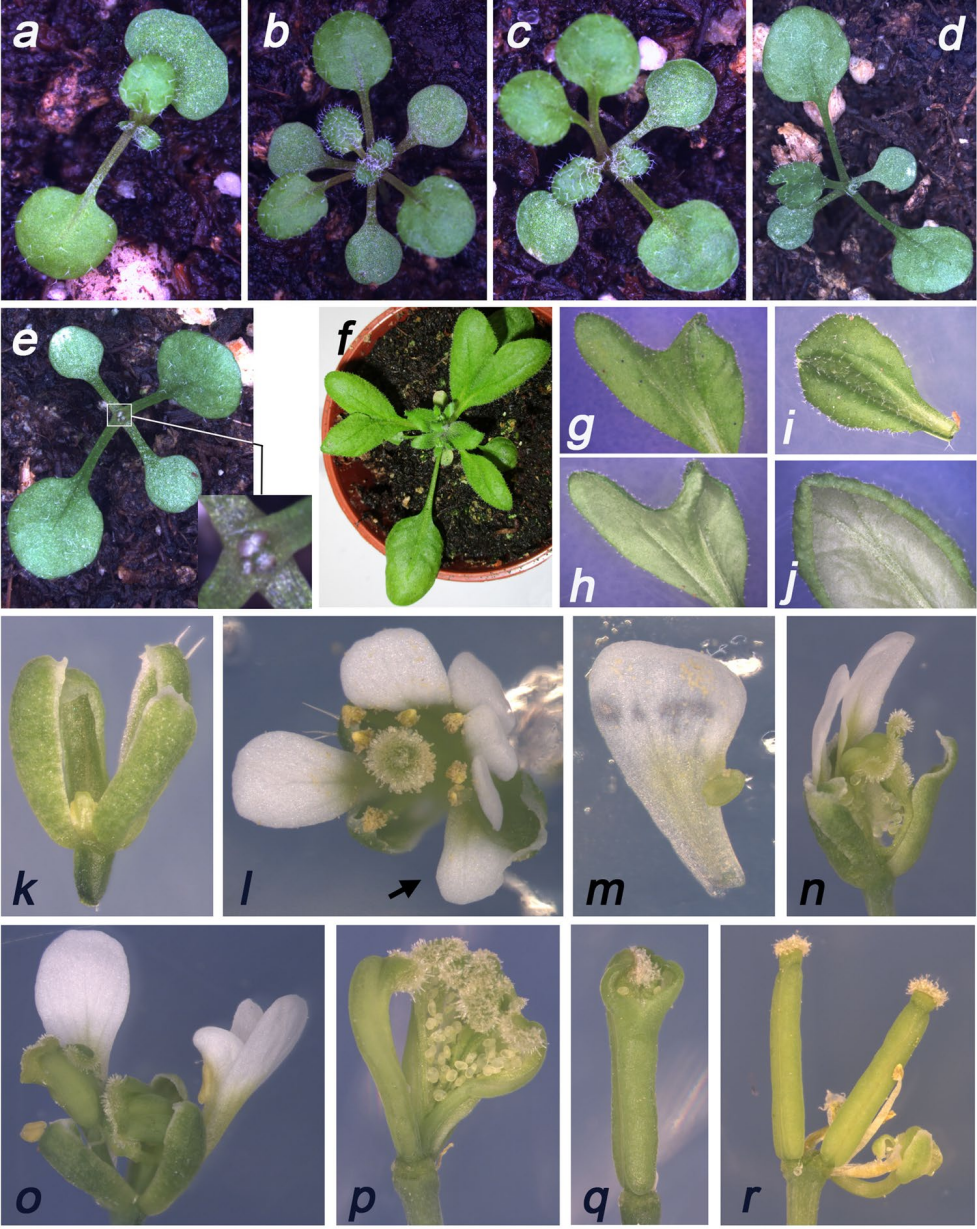
DEX-dependent DT-A cell-ablation phenotypes. **a**–**c** Cotyledon defects in seeds harvested from DEX-treated *DRNL::LHG4GR/DT-A* inflorescences. Seedlings with fused cotyledons (**a**), three cotyledons (**b**), or a bifurcation in one of the two-leaf primordia initiated in the embryo (**c**). **d**, **e** Arrested shoot apical meristems in *DRNL::LHG4GR/DT-A* seedlings germinated on DEX-containing medium. A bifurcation of leaf 3 is visible in **d** and rudimentary leaf primordia are evident in the close-up included in **e**; a corresponding wild-type SAM would be covered by successive leaf primordia similar to that shown in **b**. **f** A similar developmental arrest is observed in seedlings sprayed with DEX at the four-leaf stage; two bifurcated leaves and a compact inflorescence shoot are visible. **g**–**i** Details of representative leaf defects independent of the timepoint of DEX induction: **g**, **h** apical splitting along the mid-rib; the splitting towards the leaf base is less evident in the adaxial (**g**) than in the abaxial view (**h**). **i**, **j** Compensatory invaginations of tissues resulting from cell ablations are frequent along the mid-rib (**i**) or at the leaf margins (**j**); note the apical splitting of the mid-rib in **j**. **k**–**r** Exemplary cell-ablation phenotypes in *DRNL::LHG4GR/DT-A* flowers induced by DEX in inflorescence shoots after the opening of the first flower. **k** Ablation of organs in the inner three floral whorls. **l** Supernumerary petals and/or petaloid sepals. **m** A stamenoid petal. **n** The absence of organs in different floral whorls and misregulated carpel development. Extra carpels (**o**) and unfused carpels with ovules evident (**p**). A more subtle ablation phenotype restricted to the apical style domain (**q**) or to multiple gynoecia from a fused or bifurcated flower (**r**)

The DEX-dependent cell-ablation phenotypes in transgenic *DRNL::GRLhG4/DT-A* lines were consistent with *DRNL* promoter activity and clonal GUS sectors. To analyse the effect of ablating *DRNL* expression domains in tissue that develops embryogenically, primary inflorescences were treated with DEX and seedlings germinating from the resulting seeds were analysed. These showed single cotyledons and cotyledon fusions (Fig. 5a) or three cotyledons (Fig. 5b). When *DRNL::GRLhG4/DT-A* seedlings were germinated on DEX-containing medium, bifurcations were observed in the primary leaves (Fig. 5c) or subsequent leaves (Fig, 5d). The development of leaf primordia at the SAM was arrested (Fig. 5d, e) and subsequently resumed. A developmental arrest was similarly observed when seedlings were sprayed with DEX later in development, which affected late leaves and bolting (Fig. 5f).

Leaf bifurcation was the most frequent phenotype observed. The leaf laminas were often deeply split (Fig. 5f) and developed asymmetrically (Fig. 5g), due to developmental distortion resulting from torsional folds or hyperplasia, central invaginations, or marginal curling (Fig. 5g–j). These bifurcations along the mid-rib correlated with clonal apical and mid-rib sectors, but no larger invaginations at the leaf margins where *DRNL* was expressed in hydathodes were observed. No major differences were observed in vegetative and cauline leaf development when *DRNL::GRLhG4/DT-A* seedlings were treated with DEX after floral induction at the 6–7 leaf stages.

The DEX-induced floral phenotypes were extremely variable and most often included the absence of floral organs; for example, the absence of all inner-whorl organs or their blocked outgrowth (Fig. 5k), or the absence of petals and/or stamens (Fig. 5n, o). Some flowers showed supernumerary organs (Fig. 5l). Floral-whorl architecture was often distorted, and some organs showed homeotic transformations such as petaloid sepals (Fig. 5l) or stamenoid petals (Fig. 5m). The most frequent floral organ phenotype was tissue proliferation within the carpels, which were often highly distorted and partially open (Fig. 5n–q) or were wild type and supernumerary (Fig. 5r).

### Loss of *DRNL* function leads to defects in cotyledon vasculature

**Fig. 6:**
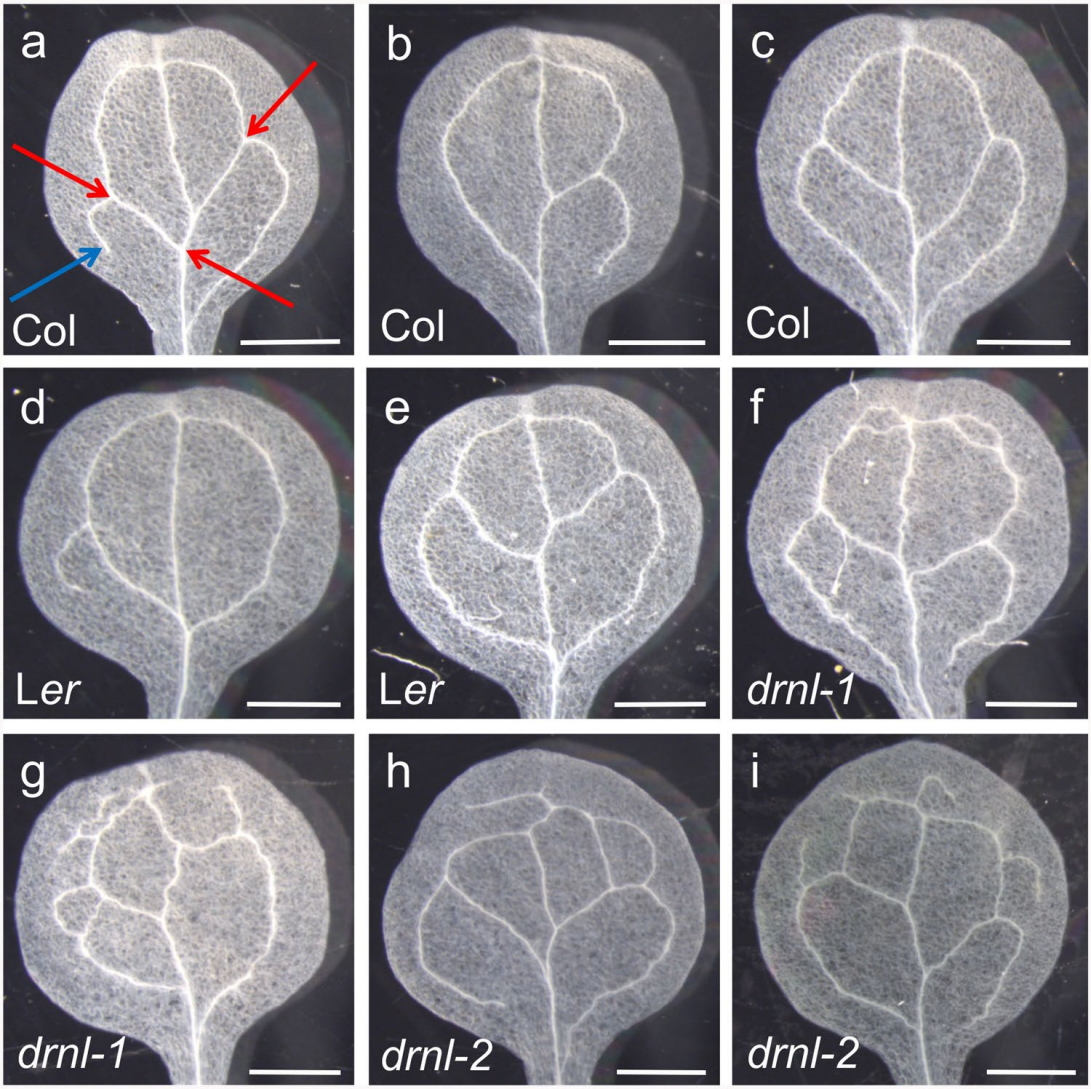
The three parameters of cotyledon venation complexity: an areole is outlined in green, and branch points are marked by red arrows and freely ending veins by blue arrows (**a**). Representative Col cotyledons with a simple (**b**) or more complex (**c**) venation pattern. Cotyledons of L*er*, showing a simple (**d**) or more complex (**e**) venation pattern. Cotyledons of *drnl-1* (**f**, **g**) and *drnl-2* (**h**, **i**). Scale bar = 0.5 mm

Expression of *DRNL::GUS* in the vasculature revealed by sector analysis and vasculature phenotypes in the ablation approach prompted us to reanalyse the vasculature phenotypes of *drnl-1* and *drnl-2* leaves and cotyledons. The leaf venation pattern of *drnl-2* was not significantly different from that of L*er* wild type (data not shown), but was clearly affected in cotyledons. We quantified the vasculature in these tissues according to three parameters: the number of areoles, branch points, and freely ending veins (Fig. 6a). Cotyledons of wild-type Col (Fig. 6b, c) and L*er* (Fig. 6d, e) seedlings showed between two and four areoles, with up to three freely ending veins and up to seven branch points. However, the venation pattern of cotyledons of *drnl-1* (Fig. 6f, g) and *drnl-2* (Fig. 6h, i) displayed a highly significant increase in venation complexity according to all three parameters (Table 2), with up to 7 areoles, 6 freely ending veins, and 13 branch points. The venation pattern of cotyledons of mutants of *DORNRÖSCHEN* (*DRN*), the *DRNL* paralogue, did not differ from that of Col (Table 2). Because *DRNL* and *DRN* function redundantly in embryonic patterning, we also analysed the venation complexity of cotyledons of *drn drnl-1* double mutants, which was the same as that of single *drnl-1* or *drnl-2* mutants, demonstrating that *DRN* and *DRNL* do not redundantly regulate cotyledon venation. Because auxin regulates venation, the venations parameters were analysed in the *pid-2* allele and did not significantly differ from wild-type L*er* values (Table 2).

**Table 2:** Cotyledon vasculature parameters for different genotypes

Genotype	Number of areoles	Number of branch points	Number of freely ending veins
Col	2.84 ± 0.72	5.71 ± 1.10	1.17 ± 0.74
L*er*	2.85 ± 0.72	5.55 ± 1.01	1.06 ± 0.81
*drn*	2.67 ± 0.67	5.47 ± 1.05	1.26 ± 0.80
*drnl-1*	3.54 ± 0.91***	7.20 ± 1.41***	1.68 ± 1.29***
*drnl-2*	3.29 ± 0.78***	7.88 ± 1.61***	2.64 ± 1.40***
*drn drnl-1*	3.23 ± 0.89**	7.63 ± 1.61***	2.51 ± 1.53***
*pid-2*	3.70 ± 0.99	7.16 ± 1.68	1.25 ± 0.93

Values are means ± SE (*n* = 100); significant differences were assessed for each parameter for each mutant against wild-type values using Student’s *t* test. **p < 0.005; ***p < 0.0001

## Discussion

We have used two complementary methods to characterise *DRNL* expression and infer its function in addition to published classical genetic and transgenic approaches. One focus was to identify the fate of cells in which *DRNL* transcription is activated in the SAM peripheral zone, i.e., to confirm *DRNL* expression as lateral organ founder-cell (LOFC) marker. Therefore, we converted a short *DRNL* transcriptional pulse into a robust cellular trajectory using DEX-inducible CRE-recombinase activity to activate a *loxP*-flanked constitutive GUS reporter. This reporter activity remained visible during subsequent organ development and traced cellular trajectories relative to previous cellular recombination events. For comparison with wild-type *DRNL* expression, we created transgenic plants carrying a *DRNL::GUS* construct analagous to the *DRNL::erGFP* construct (Chandler et al. 2011b). In contrast to previously published *DRNL::GUS* reporter constructs with incomplete lengths of upstream promoter sequence (Ikeda et al. 2006; March-Martinez et al. 2006; Nag et al. 2007), the construct here should contain all upstream and downstream regulatory elements (Comelli et al. 2016). Alternatively, we expressed the *DT-A* toxin gene DEX-inducibly behind the *DRNL* promoter for cell ablation.

Both the efficacy of the sector and ablation approach are dependent on the developmental stage at the timepoint of DEX application and reflect its half-life of about 3–5 days (Samalova et al. 2019). Both also rely on *DRNL* promoter activity and its dynamic spatio-temporal expression pattern throughout *Arabidopsis* development (Chandler et al. 2011b). For example, DEX application to a developing inflorescence simultaneously affects flowers at different developmental stages and a first important result was the striking coincidence between the native domains of *DRNL* expression in developing organs, flowers, or leaves, and clonal GUS-stained sectors or *DT-A-*induced ablation phenotypes.

### Leaf clonal sectors confirm that *DRNL* marks leaf primordium founder cells

Strong native *DRNL::GUS* expression in leaves of young seedlings was consistent with DEX-dependent clonal sectors that encompassed the entire youngest leaflets (Fig. 2d, e). This clonal trajectory is consistent with the absence of leaves via *DRNL*-induced cell ablation and suggests that *DRNL* marks leaf primordium founder cells at the SAM periphery, which are estimated to be 5–10 per layer (Furner and Pumfrey 1992; Irish and Sussex 1992). Irrespective of whether the number of *DRNL*-expressing cells matches that of previous estimates, their ablation arrests leaf initiation. The SAM recovers function with ceasing DEX activity, i.e., reduced DT-A expression. This recovery is reminiscent of mutant alleles of the *SHOOT MERISTEMLESS* gene, which are hampered in the establishment of a functional SAM during embryogenesis, but at the seedling stage can initiate leaves from residual precursor cells (Barton and Poethig 1993; Endrizzi et al. 1996).

Later cell ablations bisected the leaf along mid-rib, and these bisections were deeper when young leaflets were exposed to DEX, which suggests that pre-vascular cells are recruited early. These bifurcations directly relate to wedge-shaped apical GUS sectors which, consistent with *DRNL* promoter activity in the young leaf mid-rib, also enabled later CRE-dependent recombination events. These were indicated by narrow mid-rib-specific GUS sectors (Fig. 2i–k) and occasionally also extended laterally (Fig. 2k). The GUS-staining pattern of one lamina half of vegetative and cauline leaves (Figs. 2f or 3f) suggests that the mid-rib or primary vascular bundle forms a boundary between lamina sides. The frequency of such sectors was rare compared to that of wedge-shaped apical sectors, and probably reflects recombination in a narrow window of early leaf morphogenesis. Under this premise, the split-leaf staining suggests that in addition to previously established cellular trajectories from the L1 and L2 tunica layers into the developing leaf (Schnittger et al. 1996), cells at lateral positions within the group of LOFCs within the SAM periphery give rise to leaf sides.

The predominance of wedge-shaped apical GUS sectors observed in leaves that were young at the time of DEX application suggests that LOFCs marked by *DRNL* activity in at the vegetative SAM periphery preferentially contribute to the apical leaf domain. Additional cells for lateral leaf domains are possibly recruited subsequently and neither *DRNL::GUS* lines nor clonal GUS sectors provided evidence that late *DRNL* promoter activity in hydathodes follow a trajectory from LOFCs. Hydathode-associated expression is thus a late aspect of *DRNL* promoter activity and GUS-positive cell clones represent CRE-mediated independent recombination events in single or multiple (see Fig. 3v) cells. When induced in older leaf primordia, such marginal GUS sectors either preferentially reside or are larger in size at the leaf base, consistent with the basipetal mode of leaf maturation. The cessation of cell divisions starts at the apical tip and progresses basipetally during leaf ontogeny (Andriankaja et al. 2012), a programme that affects peri- and anticlinal cell divisions during apical and lateral growth in the earliest window of leaf development (Robles et al. 2010). The larger hydathode-associated sectors at the lamina base than those more apically (Fig. 3u) reflect the higher cell-division potential at the leaf base. Thus, the clonal GUS sectors and cell-ablation phenotypes are consistent with the *DRNL* transcription pattern during leaf development and confirm that *DRNL* transcription marks LOFCs specified at the SAM periphery, which are incorporated into leaf primordia where they contribute to the apical domain and mid-rib.

### Clonal sectors in flowers reflect floral organ ontogeny and dynamic spatio-temporal *DRNL* activity

Dynamic native *DRNL* expression in flowers marks founder cells of all floral organs (Chandler et al. 2011b) and the ablation of some or all floral organs confirms the association of *DRNL* expression with floral organ founder cells. Although belonging to different floral whorls, lateral stamens and petals are prepatterned by *DRNL* expression in two lateral domains in the stage 2 floral meristem (FM). By contrast, the four medial stamens derive from a ring-shaped *DRNL* expression domain early in floral stage 3 that focuses on medial stamen positions at stage 4 (Chandler et al. 2011b); therefore, the two sets of stamens derive from different morphogenetic patterns (Smyth et al. 1990). These temporal and spatial differences were reflected by reciprocal staining patterns (Fig. 4k, l) in which GUS sectors were restricted to either the two lateral stamens or the four medial ones. Because the two groups of *DRNL*-expressing cells that each resolves into founder cells for two petals and a single lateral stamen are spatially distinct and locate to opposite flanks of the FM (Chandler et al., 2011b), the staining of two lateral stamens can hardly result from a single recombination event. More probably, this pattern reflects the coincidence of independent excision events in lateral stamen LOFCs and substantiates the temporal series of lateral stamens being specified first and medial stamens thereafter. Remarkably, the carpel was not stained in combination with clonal GUS sectors in lateral stamens (Fig. 4k), whereas the carpel showed apical GUS sectors together with those in the medial stamens (Fig. 4l). This combinatorial selectivity of clonal GUS sectors perfectly depicts the spatio-temporal dynamics of *DRNL* expression in floral organ founder cells.

The ablation of *DRNL*-expressing founder cells often results in homeotic transformations such as petaloid stamens or stamenoid petals. These are rare in wild type (Chandler and Werr 2011), but frequent in *DRNL::DT-A* lines following DEX induction (Fig. 5m), which possibly relates to the proximity and/or imprecise specification of founder cells for petal and lateral stamens. Given the highly dynamic activity of the *DRNL* promoter during floral development, its potential relationship to auxin signalling (Comelli et al. 2016, 2020), and the role of auxin transport in phyllotaxy (Reinhardt et al. 2003), the observed absence of organs might relate to the ablation of founder cells and subsequent mis-specification or mis-positioning of primordia. We can also not exclude that overproliferation of founder cells to compensate for the ablated cells occurs. *DRNL* is expressed at later stages of gynoecium development in lateral regions that initiate the ovary valves. Loss of *DRNL* function affects gynoecium apical–basal polarity and a large proportion of *drnl-2* gynoecia do not develop fruits (Durán-Medina et al. 2017). The striped clonal GUS sectors along the gynoecium (Fig. 4o, p, r) and the non-fused carpels (Fig. 5n, p, q) potentially reflect early native *DRNL* expression, because developing carpels congenitally fuse and elongate to form a cylinder (Hawkins and Liu 2014).

### A novel function for *DRNL* in organ vasculature is revealed by clonal sector and ablation analysis

A putative function for *DRNL* in vasculature development was revealed by two lines of evidence. First, *DRNL::GUS* was expressed in the mid-rib of the first true leaves, and second, ablation of *DRNL*-expressing cells led to leaf bifurcations that reflected *DRNL* expression in the mid-rib. We, therefore, compared the vasculature phenotype of the cotyledons and leaves of *drnl-1* and *drn-2* mutants with that of wild-type L*er*. Leaf vasculature was not affected by the loss of *DRNL* function, but the significantly increased complexity of vasculature parameters in cotyledons suggests that wild-type *DRNL* function contributes to limiting vasculature complexity and vein branch points in these organs, independently of *DRN* function.

Local auxin synthesis by YUCCA flavonoid monooxygenases is essential for cotyledon vasculature development (Cheng et al. 2007), and the exogenous application of auxin or its transport inhibitors drastically changes leaf venation patterning (Biedroń and Banasiak 2018). The *drnl* cotyledon vasculature phenotypes suggest that *DRNL* contributes to auxin-related patterning and it is known that *DRNL* integrates genetically into auxin pathways involving *PIN1* (Chandler et al. 2011a). Cotyledons are embryonic tissue and the cotyledon fusion or supernumerary cotyledons in conditional *DRNL::DT-A* lines (Fig. 5a, b) here, reflect the functions of *DRNL* in cotyledon development (Chandler et al. 2007).

The mechanism by which DRNL regulates cotyledon vasculature potentially involves members of the *SHORT INTERNODES/STYLISH* (*SHI*/*STY*) gene family. The expression of these genes overlaps with that of *DRNL* in incipient and developing cotyledon and leaf primordia, in leaf apices and hydathodes (Eklund et al. 2011; Baylis et al. 2013). *STY1* activates auxin biosynthesis by transcriptionally activating *YUC4* and *YUC8* (Sohlberg et al. 2006; Eklund et al. 2010; Ståldal et al. 2012); furthermore, *SHI*/*STY* expression is feedforward-regulated by auxin (Baylis et al. 2013). Similar to *drnl* cotyledons, those of some *shi*/*sty* single and multiple mutants show an increased frequency of freely ending veins and vascular disconnections (Baylis et al. 2013). *DRNL* functions upstream of *STY*/*SHI* genes, and transcription of *STY1* and other *SHI/STY* family members are upregulated by *DRNL* induction (Eklund et al. 2011) and STY1 is also a direct *DRNL* target (Ikeda et al. 2006). Therefore, cotyledon venation is patterned in part by a regulatory cascade that includes DRNL and STY/SHI transcription factors and integrates into auxin pathways. The absence of a leaf venation phenotype in *drnl* suggests that leaf venation might be more redundantly regulated than in cotyledons; however, the ablation of the tips of the leaves along the mid-vein reflects *DRNL* expression in the mid-vein and at the tips of leaf primordia. Furthermore, *DRNL* expression in the hydathodes is relevant, because hydathodes are open vein endings connected to small parenchyma cells that typically form at the tips of leaf serrations (Candela et al. 1999), in response to YUC-mediated auxin biosynthesis (Wang et al. 2011).

Although much is known about positional information provided by auxin for lateral organ outgrowth, more precise knowledge concerning its role in the timing of floral organ specification and commitment remain scarce (Chandler 2012); therefore, indirect methods such as sector analysis that can inform the earliest stages of lateral organ initiation are innovative.

In conclusion, we have demonstrated the efficacy of using *CRE/loxP*-specific recombination based on the cell-type-specificity of *DRNL* promoter to generate GUS-stained sectors that visualise clonal lineages after cellular recombination events, and to induce the cell-type-specific ablation of *DRNL*-expressing cells. Sector analysis showed that *DRNL*-expressing cells are incorporated into lateral organ primordia and the early ablation of *DRNL*-expressing cells blocks primordium initiation. Both results confirm that transcriptional activation of *DRNL* in groups of cells in the SAM or IM peripheral zones identifies cells fated to develop into new lateral organs, thereby confirming that *DRNL* expression is a marker for LOFC specification. The variety of clonal sectors reflects the known ontogeny of organs and can be extrapolated to the *DRNL* expression pattern during the organ development. Particularly in flowers, sectors can be informative in distinguishing temporal asynchrony in floral organ initiation, for example between lateral and medial stamens. Finally, the correlation between *DRNL* transcription in the mid-rib of young vegetative leaves and the predominance of clonal GUS sectors and cell-ablation phenotypes that include the mid-rib or primary vascular bundle prompted us to revisit the phenotype of *drnl* mutant alleles and led to the identification of an undescribed cotyledon vascular phenotype.

The conclusions that can be drawn concerning patterns of *DRNL* expression and function extend beyond those reached to date from static reporter gene imaging, in situ analyses, and genetic approaches and the methodologies here represent a potentially useful adjunct to conventional methods of transcription-factor function.

#### *Author contribution statement*

WW and DG conceived and designed research. DG and PC conducted experiments. WW and DG analysed data. WW and JWC wrote the manuscript. All authors read and approved the manuscript.

## Abbreviations

DEX: Dexamethasone
DRNL: DORNRÖSCHEN-LIKE
DT-A: Diphtheria toxin A chain
LOFC: Lateral organ founder cell
SAM: Shoot apical meristem
